# A rare case of multiple paragangliomas in the head and neck, retroperitoneum and duodenum: A case report and review of the literature

**DOI:** 10.3389/fendo.2022.1054468

**Published:** 2023-01-10

**Authors:** Shin Kawanabe, Takuyuki Katabami, Ryuichi Oshima, Nobuyuki Yanagisawa, Masakatsu Sone, Noriko Kimura

**Affiliations:** ^1^ Division of Metabolism and Endocrinology, Department of Internal Medicine, St. Marianna University Yokohama Seibu Hospital, Yokohama, Japan; ^2^ Division of Metabolism and Endocrinology, Department of Internal Medicine, St. Marianna University School of Medicine, Kawasaki, Japan; ^3^ Department of Gastroenterological and General Surgery, St. Marianna University Yokohama Seibu Hospital, Yokohama, Japan; ^4^ Department of Pathology, St. Marianna University School of Medicine, Kawasaki, Japan; ^5^ Department of Clinical Research, and Department of Diagnostic Pathology, National Hospital Organization Hakodate Hospital, Hakodate, Japan

**Keywords:** catecholamine-secreting tumor, duodenal paraganglioma, head and neck paraganglioma (HNPGL), hereditary paraganglioma, multiple paragangliomas, succinate dehydrogenase B mutation

## Abstract

Pheochromocytomas and paragangliomas (PGLs) are rare non-epithelial neuroendocrine neoplasms of the adrenal medulla and extra-adrenal paraganglia respectively. Duodenal PGL is quite rare and there are only two previous reports. Herein, we report a case of multiple catecholamines (CAs)-producing PGLs in the middle ear, retroperitoneum, and duodenum, and review the literature of duodenal PGLs. A 40-year-old man complained right-ear hearing loss, and an intracranial tumor was suspected. Magnetic resonance imaging of the head revealed a 3-cm mass at the right transvenous foramen, which was surgically resected following preoperative embolization. The pathological diagnosis was a sympathetic PGL of the right middle ear. Six years later, family history of PGL with germline mutation of succinate dehydrogenase complex iron sulfur subunit B, SDHB: c.268C>T (p.Arg90Ter) was clarified. The patient had elevated levels of plasma and urine CAs again. Abdominal computed tomography scanning revealed two retroperitoneal tumors measuring 30-mm at the anterior left renal vein and 13-mm at near the ligament of Treitz. The larger tumor was laparoscopically resected, but the smaller tumor was not identified by laparoscopy. After the operation, the patient remained hypertensive, and additional imaging tests suggested a tumor localized in the duodenum. The surgically resected tumor was confirmed to be a duodenal PGL. After that, the patient remained hypertension free, and urinary levels of noradrenaline and normetanephrine decreased to normal values. No recurrence or metastasis has been found at 1 year after the second operation. CAs secretion from PGLs in unexpected location, like the duodenum of our patient, may be overlooked and leads to a hypertensive crisis. In such cases, comprehensive evaluation including genetic testing, fluorodeoxyglucose-positron emission tomography scanning, and measurement of CAs will be useful for detecting PGLs. Most previous reports on duodenal PGL were gangliocytic PGL which has been renamed composite gangliocytoma/neuroma and neuroendocrine tumor, and defined the different tumor from duodenal PGL. We reviewed and discussed duodenal PGLs in addition to multiple PGLs associated with SDHB mutation.

## 1 Introduction

Pheochromocytomas and paragangliomas (PPGLs) are rare non-epithelial neuroendocrine neoplasms of the adrenal medulla or the extra-adrenal paraganglia among the sympathetic or parasympathetic chain, respectively (1, 2). Paragangliomas (PGLs) account for 10–20% of all patients with PPGLs (3), but gastrointestinal PGLs are extremely rare. Although duodenal PGLs have been thought the most common form of gastrointestinal PGLs, most cases previously reported were likely gangliocytic PGLs (4, 5). The 2022 WHO classification of paragangliomas and pheochromocytomas clearly defined that PGLs are distinctively different from gangliocytic PGLs. PGLs are composed of spindle (sustentacular) cells and round or polygonal epithelioid cells arranged in a “Zellballen” pattern, and they produce catecholamines (CAs). In contrast, gangliocytic PGL contains mixtures of three distinct histological patterns: typical neurofibroma (with proliferating neuritis and Schwann cells), ganglion cells mixed with Schwann cells, and proliferation of clear epithelioid cells arranged in clusters or radial patterns resembling carcinoid tumors, and they do not produce CAs. However, the two tumors have been described in previous studies as similar diseases (2, 4). Thus, the true number of patients with duodenal PGLs is much less than previously reported.

Furthermore, production of CAs has not been evaluated in the vast majority of patients with gastrointestinal PGLs (6). Lack of clinical suspicion and unrecognized hypersecretion of CAs may lead to hypertensive crisis (6–8). We herein report our experience with multiple CAs-producing PGLs in the duodenum, middle ear, and extra-adrenal retroperitoneum of a patient along with a review of the literature.

## 2 Case presentation

**Figure 1:**
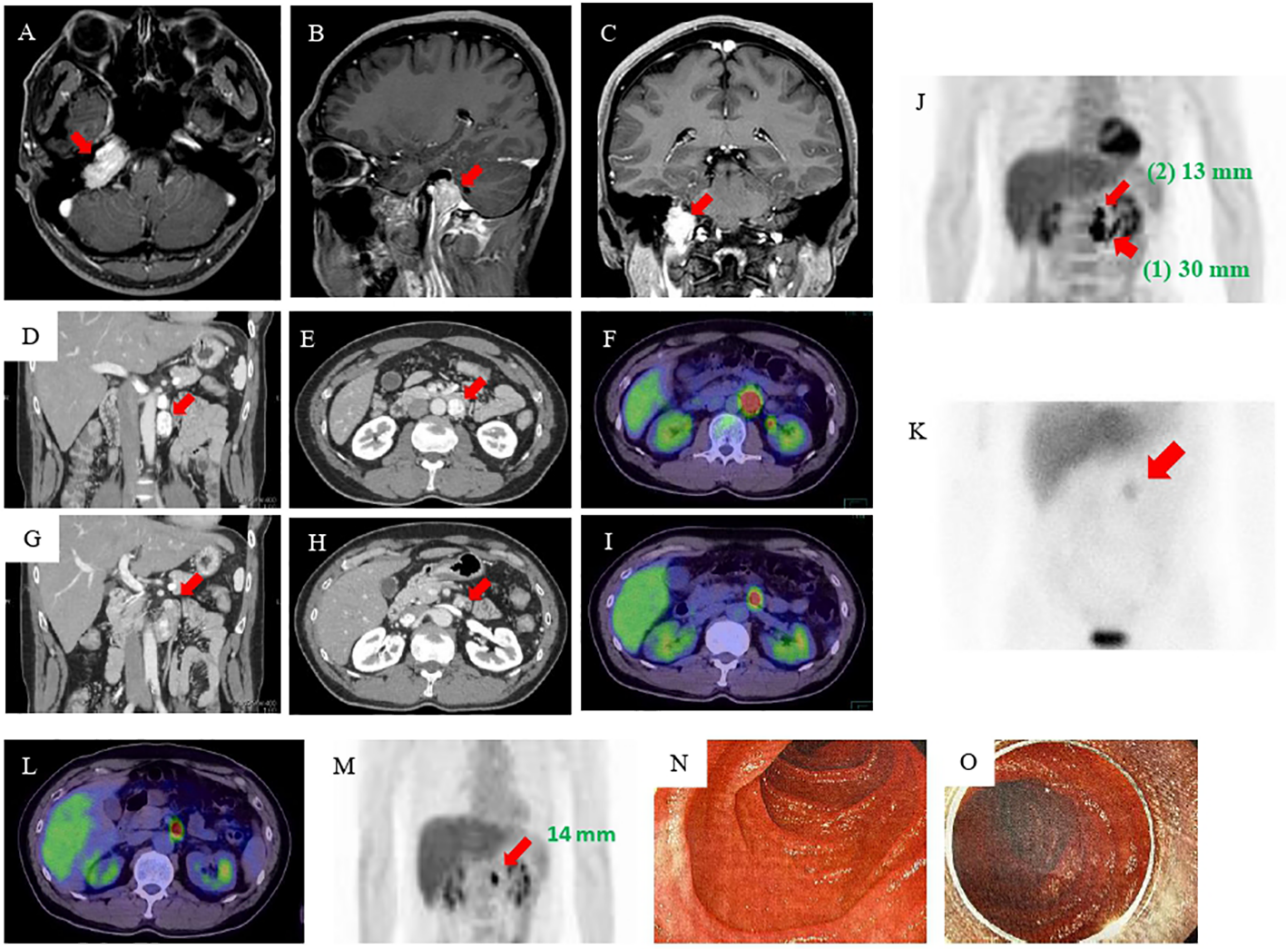
MRI, CT, 18F-FDG-PET, and MIBG imaging results. **(A–C)** MRI of the head revealed an approximately 3-cm mass at the right transvenous foramen. **(D, E)** Enhanced abdominal CT revealed two tumors in the retroperitoneum: a nodal tumor measuring 30 mm (arrow) on the anterior left renal vein and **(G, H)** a tumor measuring 13 mm (arrow) near the ligament of Treitz. **(F, I, J)** 18F-FDG-PET showed accumulation in both tumors [(1) the tumor measuring 30 mm (wide arrow) and (2) the tumor measuring 13 mm (narrow arrow)]. **(K)** MIBG scintigraphy showed accumulation in an area consistent with a mass (arrow). **(L, M)** Esophagogastroduodenoscopy and enteroscopy did not identify the smaller tumor. **(N, O)** MRI, magnetic resonance imaging; CT, computed tomography; FDG-PET, fluorodeoxyglucose-positron emission tomography; MIBG, 123I-metaiodobenzylguanidine.

A 40-year-old man presented to our affiliated hospital with right-ear hearing loss. He had no notable medical history. Audiometry revealed hearing loss in the right ear, but otoscopy showed no abnormalities in the auditory canal or tympanic membrane. The presence of an intracranial tumor was therefore suspected, and imaging studies were performed. Magnetic resonance imaging of the head revealed an approximately 3-cm mass at the right transvenous foramen (**Figures 1A–C**). In response to this finding, the tumor was resected in the neurosurgery department of our affiliated hospital. At that time, the tumor was suspected to be a PGL, and it was removed after preoperative embolization. The pathological diagnosis was PGL of right middle ear (**Figure S1**). Because the patient complained no headaches or hypertension, the 24-h urine CAs analyses were not measured.

The patient’s postoperative course was good, and he was followed up at the same hospital by imaging inspection only. Then, six years later, his eldest daughter was admitted to another hospital with hypertensive crisis caused by a retroperitoneal PGL. In addition, the fact that his twin brother had died owing to a hypertensive crisis when he was 23 years old came to light (**Figure S2**). This prompted his physician to perform further examinations. Elevated levels of plasma and urine CAs were found, and abdominal magnetic resonance imaging (MRI) revealed a 3-cm retroperitoneal tumor. He was suspected of having PPGLs and was referred to our hospital for further examination. The results of adrenal hormone tests reevaluated in our hospital were as follows: adrenaline <0.01 ng/mL (reference: 0–0.17 ng/mL), noradrenaline 2.0 ng/mL (reference: 0.15–0.50 ng/mL), dopamine <0.02 ng/mL (reference: 0–0.03 ng/mL), urinary adrenaline 2.3 µg/day (reference: 1.1–22.5 µg/day), urinary noradrenaline 1310 µg/day (reference: 29.2–118 µg/day), urinary metanephrine 0.06 mg/day (reference: 0.05–0.20 mg/day), and urinary normetanephrine 2.3 mg/day (reference: 0.1–0.28 mg/day). An enhanced abdominal computed tomography (CT) scan revealed the presence of tumors in the area of the retroperitoneum: a 30-mm nodal tumor anterior on the left renal vein (**Figures 1D–F**) and a 13-mm tumor near the ligament of Treitz (**Figures 1G–I**). There were no abnormally enlarged retroperitoneal lymph nodes, and no obvious abnormalities were observed in the liver, gallbladder, spleen, kidney, and gastrointestinal tract. ^18^F-fluorodeoxyglucose-positron emission tomography (FDG-PET) showed FDG accumulation in these tumors (**Figure 1J**). ^123^I-metaiodobenzylguanidine (MIBG) scintigraphy also showed an accumulation in an area consistent with a mass, but because of the close proximity of the two tumors, the hormone production of the smaller tumor could not be identified (**Figure 1K**).

Based on these results, he was diagnosed as having abdominal PGLs. We considered that the tumors resided in the retroperitoneum and therefore attempted laparoscopic removal of both of them. We completely removed the 30-mm tumor. However, we were unable to identify the 13-mm tumor, and we finished the operation. The resected tumor was encapsulated. Hematoxylin-eosin staining showed a Zellballen structure composed of chief cells and sustentacular cells that is typical for PGLs (**Figure S3**). The immunohistochemical staining studies indicated that the tumor was positive for chromogranin A, tyrosine hydroxylase, and dopamine β-hydroxylase but were negative for succinate dehydrogenase complex iron sulfur subunit B (SDHB), and choline acetyltransferase (ChAT). The grading system for adrenal pheochromocytoma and paraganglioma (GAPP) score was 5 points, suggesting a tumor with moderate-grade for malignancy (**Table 1**) (9). Pathological investigation confirmed the diagnosis of retroperitoneal PGL (**Figure S3**). Genetic testing identified a known *SDHB* germline mutation, SDHB: c.268C>T (p.Arg90Ter) (10) identical to that of the daughter. However, despite the resection of the retroperitoneal PGL, the patient remained hypertensive, and his urinary CAs levels were twice the upper limit of normal at two months after the operation.

**Table 1:** GAPP score for each paraganglioma.

Tumor location	Duodenum	Retroperitoneum	Middle ear*
Histology: Zellballen pattern	0	0	0
Cellularity	0 (under 150/U)	1 (150–250/U)	Not assessable
Comedo necrosis	0 (absent)	0 (absent)	0 (absent)
Ki67 labeling index (%)	1 (1.3%)	2 (5%)	1 (2%)
Capsular/vascular invasion	0 (absent)	1 (present)	Not assessable
CA type: Norepinephrine type	1	1	Not assessable
Total score	2	5	Not assessable
Histological grading	Well	Moderate	Not assessable

*The middle ear PGL was resected after preoperative embolization, and the GAPP score was not available due to artificial degeneration.

GAPP, grading system for adrenal pheochromocytoma and paraganglioma; U, unit; CA, catecholamine; PGL,s paraganglioma.

Therefore, we performed additional imaging studies. ^123^I-MIBG scintigraphy showed no accumulation, but ^18^F-FDG PET/CT and a contrast-enhanced CT study revealed that the tumor near the ligament of Treitz was still present and had become mildly enlarged. He was admitted for re-evaluation one year after the surgery. As the residual tumor was not visible during the previous laparoscopic operation, we considered the tumor to be in the duodenum lumen rather than in the retroperitoneum. Although he underwent esophagogastroduodenoscopy, endoscopic ultrasonography, and enteroscopy, the tumor still could not be identified (**Figures 1L–O**). On the basis of the CT findings, we thought that the tumor was a duodenal PGL and performed an open duodenectomy.

**Figure 2:**
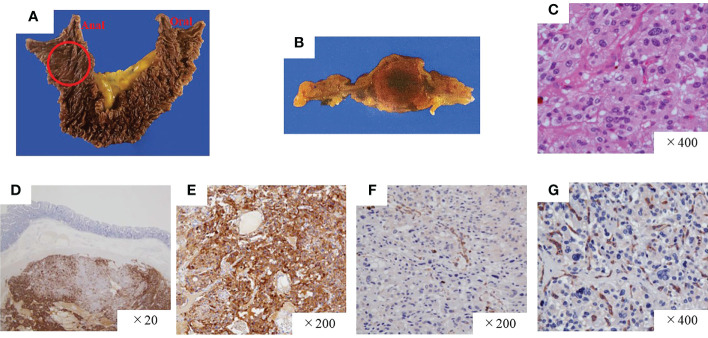
Pathological findings of the paraganglioma of the duodenum. **(A, B)** Gross findings of the duodenal tumor. **(C)** Hematoxylin and eosin staining, high-power field. The tumor was positive for chromogranin A **(D)** and tyrosine hydroxylase **(E)** but negative for SDHB **(F)** and ChAT **(G)**. SDHB, succinate dehydrogenase complex iron sulfur subunit B; ChAT, choline acetyltransferase.

A pathological investigation confirmed that a 10-mm sized submucosal tumor located at the junction between the horizontal portion of the duodenum and the jejunum. Histologically, the tumor was mainly located in the submucosal to subserosal layer (**Figure 2**). The tumor cells with vacuolated cytoplasm and hyperchromatic nuclei showed Zellballen patterns, associated with well-developed vessels. The immunohistochemical studies were positive for chromogranin, tyrosine hydroxylase, and dopamine β-hydroxylase and negative for ChAT supporting the diagnosis of a sympathetic PGL. The Ki67 marker showed a low mitotic index of 1.3%. SDHB immunostaining showed loss of reactivity in both duodenal and retroperitoneal PGLs. The pathological results of the middle ear, retroperitoneal, and duodenal tumors are summarized in **Table 1**. After surgery, the patient’s blood pressure remained within the normal range without medications, and urinary levels of noradrenaline and normetanephrine decreased to 51.2 µg/day and 0.18 mg/day, respectively. The tumor had disappeared on imaging. No recurrence or metastasis has been observed at 1 year from surgery.

## 3 Discussion

PPGLs are rare non-epithelial neuroendocrine neoplasms found within the adrenal medulla or the extra-adrenal paraganglia among the sympathetic or parasympathetic chain (1, 2). Gangliocytic PGLs occasionally have been misinterpreted as several unusual lesions of PGL because the tumors include sustentacular cells that are one of the characteristic features in PGLs. However, the most recent WHO classification of PPGL clearly shows that duodenal PGLs and gangliocytic PGLs are distinctively different tumors (2). Thus, to review patients with definitive duodenal PGL, we performed a Medline search for PGLs and gangliocytic PGLs of the duodenum previously reported in the English literature (4, 11–18). Based on our strict review of the clinicopathological findings of each report, we identified only two reports describing a definite diagnosis of duodenal PGL (**Table 2**) (17, 18). Furthermore, we found that the present case appears to be the first to show CA-producing duodenal PGLs expressing various enzymes involved in CA biosynthesis and postoperative normalization of plasma and urinary CA concentrations.

**Table 2:** Cases of duodenal paraganglioma.

Case (Reference, issue year)	Age/Sex	Main complaint	Tumor size	Presence of CA secretion	SDHB-IHC
1 ( (17), 2006)	68/M	N/D	N/D	N/D	N/D
2 ( (18), 2022)	17/F	Refractory tarry stool	40 mm	N/D	Negative
Present case	47/M	No symptoms caused by CA	14 mm	Noradrenaline secretion	Negative

CA, catecholamine; SDHB-IHC, succinate dehydrogenase complex iron sulfur subunit B immunohistochemistry; N/D, not described.

An interesting aspect of this patient is the masquerading of the duodenal PGL as a retroperitoneal mass on the various imaging modalities. Although we initially thought that two tumors existed in the retroperitoneum before the second operation, only a solitary tumor could be found in the space at surgery. Unfortunately, overproduction of CAs remained, and one of the two tumors could still be visualized on ^18^F-FDG-PET CT examination after the surgery (**Figure 1M**). Hence, following the results of the imaging examinations, the patient underwent enteroscopy and endoscopic ultrasonography to identify lower duodenal and/or upper jejunal PPGLs, but no lesion in the intestinal lumen could be detected. However, because it was conceivable that the tumor could be located in the submucosa of the small intestine, such as an “epithelial neuroendocrine tumor, e.g. carcinoid,” we performed an open duodenectomy, and histopathological examinations indicated the presence of a duodenal submucosal PGL. Thus, if retroperitoneal PGLs are encountered, the possibility that some of them might be intestinal in origin should be considered. In addition, when catecholamines remain high but no primary site is found, especially in cases of *SDHB* mutations, we must be careful to look for sites other than the usual site of PGL occurrence, such as the duodenum.

In the present patient, ^123^I-MIBG scintigraphy showed no accumulation in the duodenal PGL. Previously, ^123^I-MIBG was the most widely used diagnostic modality, but several reports have indicated that the examination shows poor resolution and a lower sensitivity for small tumors (19), extra-adrenal lesions, metastatic sites, and patients with *SDHx* mutation (20, 21). One possible explanation for the discrepancy between MIBG accumulation in the duodenal versus retroperitoneal PGL in this patient is the difference in mass size because the two tumors must both be harboring the *SDHB* mutation, can be classified as extra-adrenal PGLs, and seem to be non-metastatic. Recent guidelines have recommended FDG-PET-based nuclear modalities as the first option for identifying tumor localization in patients with PPGLs (20). In accordance with this statement, the smaller duodenal PGL in our patient was visualized on ^18^F-FDG-PET/CT. In summary, higher-resolution imaging techniques, such as ^18^F-PET/CT and contrast-enhanced CT, should be selected to determine tumor localization and staging of PPGLs, especially in patients with masses smaller than 10 mm (19–21). Endoscopic ultrasound is useful for detecting PGLs in the esophagus, stomach, bulbar and descending portions of the duodenum. PGLs at these sites must be detectable during the examination. However, because the lesion in our patient was located horizontally to the ascending portion of the duodenum (near the ligament of Traits), it could not be observed on the endoscopic examination.

Head and neck PGLs (HNPGLs) generally arise from the parasympathetic ganglia located along the glossopharyngeal and vagal nerves in the neck and base of the skull and are recognized as non-CAs producing tumors. Recent study of immunohistochemistry of ChAT, an enzyme involved in acetylcholine synthesis, demonstrated that most head and neck PGLs positive for ChAT, and designated HNPGLs as acetylcholine-producing parasympathetic tumors (22). However, HNPGLs can occasionally arise from the cervical sympathetic chain with CAs over-secretion (23). Immunohistochemical analysis in our patient clearly showed that the HNPGL synthesized CAs but acetylcholine (**Figures 2**, **S1**, **S3**). Rijken et al. indicated a higher risk of CAs overproduction in patients with HNPGLs harboring an SDHB germline mutation (24). Our case is a typical example of a sympathetic HNPGL harboring an SDHB germline mutation. However, we did not confirm whether the tumor released CAs into the bloodstream based on the presence or absence of CA-related symptoms other than hypertension at diagnosis or the hemodynamic records during the first operation. Nonetheless, we believe it is worth investigating the patient’s PGLs with comprehensive assessments, including an assay for CA metabolites, radioactive scanning, family history, and genetic testing after the first operation. Currently, nearly 40% of all HNPGLs are recognized as hereditary (23).

Although our present case is extremely rare, efforts that enhance awareness of PGLs among non-endocrinologists (e.g., neurosurgeons, otolaryngologists, and gastroenterologists) are pivotal in their recognition. The efforts should lead to avoid the occurrence of hypertensive crisis in the perioperative period, overlook patients with multiple PPGLs, and provide a helpful medical information for patient’s relatives.

## 4 Conclusion

We present the first case, to our knowledge, of a duodenal PGL producing CAs that was completely verified by analysis of enzyme expression involved in the biosynthesis of CAs and postoperative normalization of the concentrations of the CAs. The secretion of CAs from a PGL arising in an unexpected location, such as the duodenum in the present patient, may be overlooked. The lack of clinical suspicion and unrecognized hypersecretion of CAs from such tumors may lead to a hypertensive crisis. Because PGLs can occur in various organs, efforts that enhance awareness regarding PGLs among non-endocrinologist are pivotal in their recognition. Further, if a physician suspects the presence of PGL, a comprehensive evaluation including genetic testing, FDG-PET scanning, and measurement of CAs will be useful in identifying the disorder.

## Data availability statement

The original contributions presented in the study are included in the article/**Supplementary Material**. Further inquiries can be directed to the corresponding author.

## Ethics statement

Ethical review and approval was not required for the study on human participants in accordance with the local legislation and institutional requirements. The patients/participants provided their written informed consent to participate in this study. Written informed consent was obtained from the individual(s) for the publication of any potentially identifiable images or data included in this article.

## Author contributions

SK and TK are the co-first authors for this case report. TK is the corresponding author supervising this work. RO performed the surgery. SK and TK were monitoring the patient’s post-operative progress. NY and NK made the pathological diagnosis. SK, TK, and NK performed analysis on all data interpretation from the literature review and drafted the manuscript. All authors read and approved the final manuscript.
