# Supplementary figures and images for: A rare case of multiple paragangliomas in the head and neck, retroperitoneum and duodenum: A case report and review of the literature
Source: Front Endocrinol (Lausanne). 2023 Jan 10;13:1054468. doi: 10.3389/fendo.2022.1054468 (PMC9871575; doi:10.3389/fendo.2022.1054468)

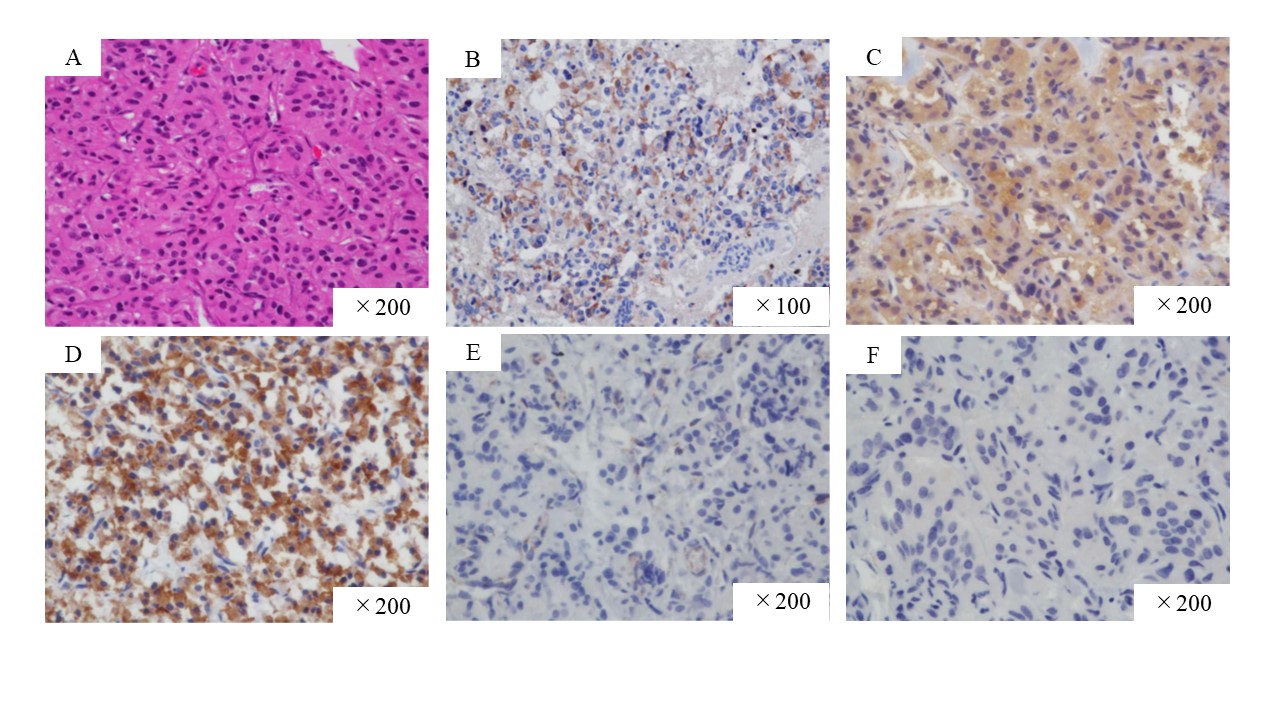

Supplement: Supplementary file 1 [file Image_1.jpeg]

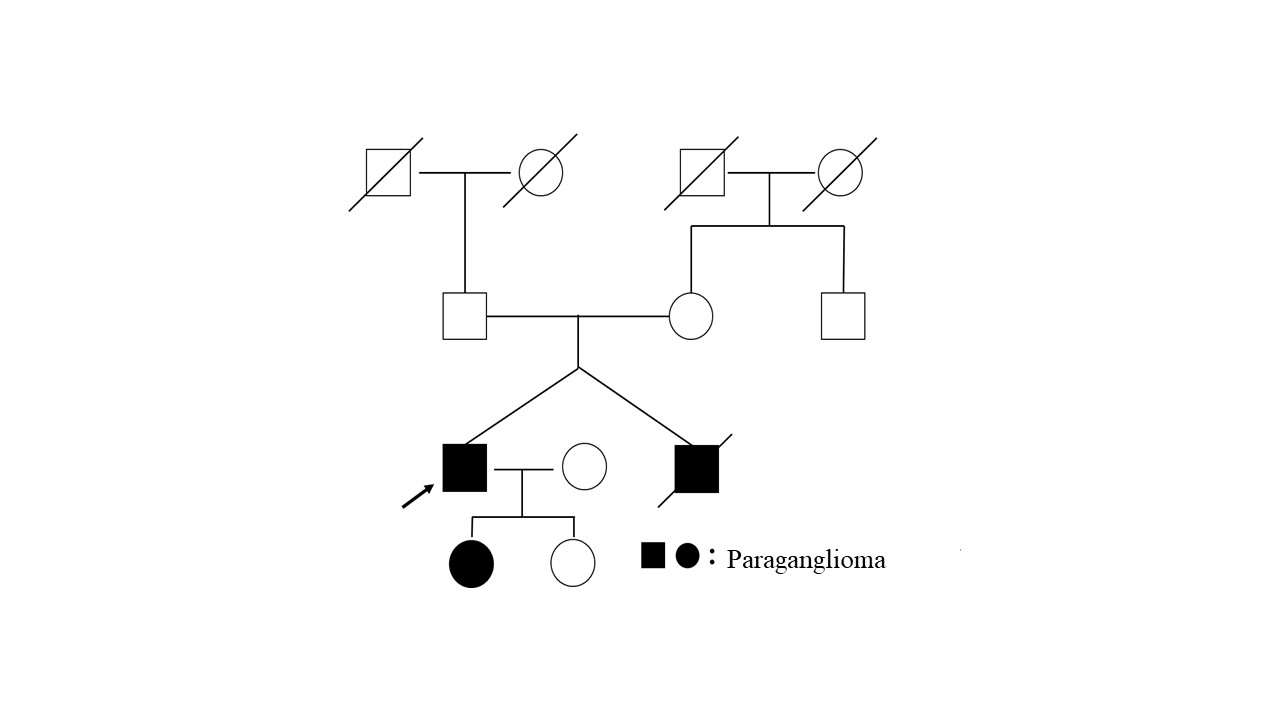

Supplement: Supplementary file 2 [file Image_2.jpeg]

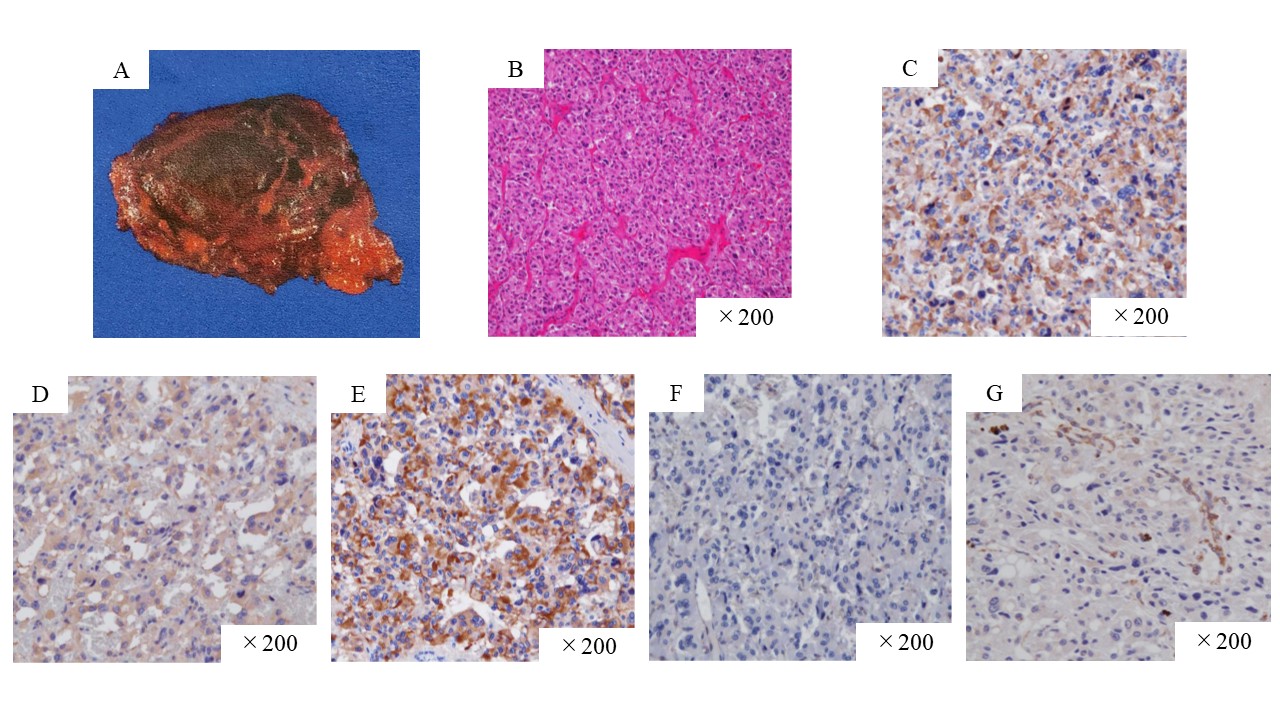

Supplement: Supplementary file 3 [file Image_3.jpeg]
